# Pathway Projector: Web-Based Zoomable Pathway Browser Using KEGG Atlas and Google Maps API

**DOI:** 10.1371/journal.pone.0007710

**Published:** 2009-11-11

**Authors:** Nobuaki Kono, Kazuharu Arakawa, Ryu Ogawa, Nobuhiro Kido, Kazuki Oshita, Keita Ikegami, Satoshi Tamaki, Masaru Tomita

**Affiliations:** Institute for Advanced Biosciences, Keio University, Fujisawa, Japan; Cairo University, Egypt

## Abstract

**Background:**

Biochemical pathways provide an essential context for understanding comprehensive experimental data and the systematic workings of a cell. Therefore, the availability of online pathway browsers will facilitate post-genomic research, just as genome browsers have contributed to genomics. Many pathway maps have been provided online as part of public pathway databases. Most of these maps, however, function as the gateway interface to a specific database, and the comprehensiveness of their represented entities, data mapping capabilities, and user interfaces are not always sufficient for generic usage.

**Methodology/Principal Findings:**

We have identified five central requirements for a pathway browser: (1) availability of large integrated maps showing genes, enzymes, and metabolites; (2) comprehensive search features and data access; (3) data mapping for transcriptomic, proteomic, and metabolomic experiments, as well as the ability to edit and annotate pathway maps; (4) easy exchange of pathway data; and (5) intuitive user experience without the requirement for installation and regular maintenance. According to these requirements, we have evaluated existing pathway databases and tools and implemented a web-based pathway browser named Pathway Projector as a solution.

**Conclusions/Significance:**

Pathway Projector provides integrated pathway maps that are based upon the KEGG Atlas, with the addition of nodes for genes and enzymes, and is implemented as a scalable, zoomable map utilizing the Google Maps API. Users can search pathway-related data using keywords, molecular weights, nucleotide sequences, and amino acid sequences, or as possible routes between compounds. In addition, experimental data from transcriptomic, proteomic, and metabolomic analyses can be readily mapped. Pathway Projector is freely available for academic users at http://www.g-language.org/PathwayProjector/.

## Introduction

With a long tradition of being a descriptive discovery science, the field of scientific visualization has been an integral part of biosciences and has also been an indispensable approach for understanding complex, large-scale data in molecular biology. Numerous approaches for information visualization have been successfully utilized and have contributed to the understanding of genomic information, including those for the protein 3D structure, sequence alignment, and phylogenetic trees [1]. Genome browsers, such as Gbrowse [2], UCSC Genome Browser [3], and Ensembl [4], have been a particular success because they provide a visual context [5]. Genome browsers show gene structures and their locations within the genome, and they can also be used to map novel knowledge and experimental data to display them in a genomic context. Systems biology approaches [6], [7] attempt to understand cellular processes as a system of molecular interactions. In post-genomic research, these approaches demand another context for biochemical pathways in order to understand biological information. A biochemical pathway is a series of reactions that consists of enzymes, proteins, and molecular compounds [8], and is a useful context for understanding how gene disruptions or alterations of conditions associate with a phenotype [9]. For example, in microarray or proteomic experiments, researchers can map their experimental data through pathway mapping systems, such as ArrayXPath II [10], GenMAPP [11], MEGU [12], and Pathway Explorer [13], to gain a comprehensive understanding of cellular regulation and to explore the existence of alternative pathways after gene deletions or change in conditions. Therefore, visualization approaches allow for an intuitive understanding of a large quantity of data that is inherently difficult to comprehend, while biochemical pathways provide a suitable context for observing the systematic cellular behavior that is analyzed through “-omics” experiments [14]. Pathway browsers will thus enhance systems biology research.

Most existing pathway maps have been provided as part of major public pathway databases at their websites. These maps are subdivided into individual pathways, in part due to technical limitations in manipulating large images on the World Wide Web. Given that pathways are essentially connected *in vivo* and that highly comprehensive experimental data that encompass a wide variety of pathways is readily available, arbitrary partitioning of pathways is often not useful for the mapping and observation of comprehensive experimental data. For instance, the glycolysis/gluconeogenesis pathway (map00010) in KEGG [15] links to five pathways: the citrate cycle (map00020), the pentose phosphate pathway (map00030), starch and sucrose metabolism (map00500), carbon fixation in photosynthetic organisms (map00710), and propanoate metabolism (map00640). Users have to constantly switch back and forth between the maps to observe reactions that encompass multiple pathways. Therefore, with the advancement in web development technologies [16], several pathway databases have started to release integrated pathway maps that allow comprehensive viewing. For example, the KEGG Atlas [17], iPath [18] and the new beta version of Reactome [19] display comprehensive integrated pathway maps without page transitions, which have been implemented as zoomable and scalable maps. The Omics Viewer [20] in BioCyc [21] implements this feature with pop-ups upon mouse-over action. These interface technologies that enable the continuous display of a large image at different scales without page transitions are collectively known as the Zoomable User Interface (ZUI). ZUI is successfully utilized for the representation of geographical information as typified by Google Maps (http://maps.google.com/), as well as for the visualization of gene networks and the implementation of genome browsers [22], [23], [24], [25].

Despite the recent availability of several integrated pathway maps, the abstraction level of represented entities in these maps is often not sufficient to map experimental data, which is primarily due to the objectives of each pathway database. For example, the KEGG Atlas and Omics Viewer do not show genes and enzymes as nodes, but instead represent them as reaction edges. Reactome only shows enzymes as nodes, which limits its applicability for the mapping of microarray data, since many enzymes exist as heteromers that are comprised of several proteins. Similarly, when only reactions are represented in the map, data from metabolomic experiments cannot be mapped. To the best of our knowledge, there is currently no pathway browser that can map experimental data for genes, enzymes, and metabolites simultaneously using a comprehensive integrated pathway map.

In this article, we present Pathway Projector, a pathway browser that allows the mapping of multi-omics information on an integrated pathway map through an intuitive user interface and ZUI. The integrated pathway map of Pathway Projector is based on the widely used layout of the KEGG Atlas with the addition of nodes for genes and enzymes for the mapping of experimental data from transcriptomic, proteomic, and metabolomic experiments. We also identify and discuss the requirements for an ideal pathway browser.

## Materials and Methods

### Requirement Analysis

As a result of a close collaboration between our bioinformatics group and experimental biologists, we have first identified the requirements for a pathway browser. We have analyzed the requirement especially in consideration of the current situations facing the biologists working with large-scale omics data with systems biology approaches, where they require a comprehensive understanding of cellular workings. Therefore, the pathway browser should be continuous and global, covering the omics layers of genome, transcriptome, proteome, and metabolome, while being intuitive and not requiring too many user interactions to allow rapid navigation for the day-to-day heuristic usage. The requirements are categorized in five groups: (R1) pathway representation, (R2) data access, (R3) mapping and editing, (R4) data export and exchange, and (R5) availability.

### Implementation

#### Pathway map

Several pathway maps are already commonly used by biologists. We therefore chose to utilize the layout of an existing familiar pathway map as the basis of our pathway browser rather than creating a new one. The KEGG Atlas was selected for several reasons: (1) a global integrated map is provided; (2) it is a part of one of the most popular pathway databases [26], [12], [27]; and (3) the reference pathway layout can be utilized for the representation of pathways in a wide variety of organisms. The KEGG database provides various tools and a wealth of pathway-related data that are curated with controlled identifiers and external references. These identifiers and references are useful for the implementation of many functions of the pathway browser and for the interoperability of the tool. Since the KEGG Atlas only represents metabolites as nodes per se, all related gene and enzyme nodes have been automatically added on the midpoint of reaction edges of the KEGG Atlas pathway map and a scalable vector graphics (SVG) file has been generated in Perl. To calculate the midpoint of edges, all quadratic bézier curves used for the representation of reaction edges were converted into polylines for computational efficiency. Following the automatic positioning of enzyme nodes, several nodes were manually curated using Adobe Illustrator CS3 13.0.2 for layout optimization. Enzyme nodes were partitioned into multiple compartments when several genes comprised the heteromeric enzyme, with a horizontal layout for genes ≤6 or with two rows of up to six columns for genes ≤12. A list of gene names only are shown for genes >12. As a result, the reference pathway map contains 1572 metabolite nodes and 1813 enzyme nodes. As examples of the organism specific pathways, there are 1365 gene nodes in E. coli and 2883 in human.

In order to provide the pathway browser as cross platform software without the need for user maintenance and installation, Pathway Projector is implemented as a web application using HTML and JavaScript with the AJAX (Asynchronous JavaScript + XML) web development paradigm. The Ext JS 2.0 (http://extjs.com/) library was utilized to implement the overall user interface framework. ZUI for the large pathway map is implemented by using the Google Maps API for intuitive and familiar user interface and to take advantage of many online tools that are associated with the API. Since large images need to be split up into image tiles that are 256×256 pixels for use with the Google Maps API, the original large pathway map of 8192×8192 pixels was split using the generateGMap function available in the G-language Genome Analysis Environment v.1.8.8 [28], [29], [30] to produce five zoom levels. The number of tiles increases by the power of 4 depending on the zoom level: 1 in level 0, 4 in level 1, 16 in level 2, 64 in level 3, 256 in level 4, and 1024 in the maximum zoom level 5. The pathway map has been made clickable by sending the coordinate information and asynchronously retrieving related information upon the user mouse click event, which is displayed through the InfoWindow function of Google Maps API as an information window. The information window contains the retrieved annotation of each component, including the common name, identifier, structural formula or chemical equation, and links to external databases such as KEGG, PubChem [31], ChEBI [32], and MSDchem [33] for metabolites and, ExPASy [34], MetaCyc [35], Brenda [36], IntEnz [37], PUMA2 [38], and IUBMB [39] for enzymes (see Table S1 for a complete listing for organism-specific database references). Although the default reference pathway only contains external links to enzymes, when an organism specific pathway map is opened from the “Organism Selection” tab, the information window on the nodes also shows gene-centric links to suitable databases for the organism (*e.g.* EcoGene [40], MGI [41]). Nodes within the pathway map show their corresponding labels (common names for genes and metabolites, EC number for enzymes) at zoom levels higher than 4, utilizing the semantic zooming capabilities of Google Maps API.

Organism-specific pathway maps for 843 species, including both eukaryotes and prokaryotes, were subsequently generated using the reference pathway map based on KEGG Orthology.

#### Search and data retrieval

Pathway Projector has four types of search functionalities: (1) by keyword and identifiers; (2) by molecular mass; (3) by computation of possible routes between two metabolites; and (4) by sequence similarity. Searches by keyword, identifiers, and molecular mass were established by searching through a server-side database reconstructed from KEGG flatfile distributions. The results are listed in a search result panel and are also visually highlighted by red markers placed onto the respective components on the pathway map. Additional gray polylines are drawn on corresponding reaction edges when the search result points to enzymes or genes. The red markers can be clicked to invoke an information window to display more detailed annotations. The route search and similarity search capabilities are implemented as wrappers of PathComp (http://www.genome.ad.jp/kegg-bin/mk_pathcomp_html) and KEGG BLAST Search (http://blast.genome.jp/) to take advantage of existing KEGG services. Since PathComp calculates every possible route between two given metabolites from all combinations in the KEGG database, only the paths that are available within the KEGG Atlas layout are displayed in the results. For the BLAST search, sequence type (nucleotide or protein) and the type of BLAST program (blastn, blastp, blastx, tblastx), are automatically interpreted by the system, in which sequences comprising 80% A, T, G, C, or N are considered to be nucleotide sequences. The Pathway prediction tool reconstructs the pathway from given multi-FASTA sequence files using BLAT and SwissProt [42] using the GEM System [43].

#### Mapping and editing

The Pathway mapping tool modifies the SVG map based on user input, by changing the visibility, size, color, and labels of edges and nodes, and subsequently creates an overlay. Users can also place predefined icons or any image available in the World Wide Web by URLs and show the directionality of reactions by adding arrowheads to reaction edges. When values for time-series or multiple conditions are specified for nodes, graphs or charts generated by the Google Chart API (http://code.google.com/intl/en/apis/chart/) are displayed on the nodes. Quikmaps (http://quikmaps.com/) was utilized to implement manual annotation and editing capabilities.

## Results and Discussion

### Requirement Analysis

The requirements are categorized in five groups: (R1) pathway representation, (R2) data access, (R3) mapping and editing, (R4) data export and exchange, and (R5) availability (see Table 1 for a summary).

**Table 1:** Comparison of existing pathway-related software and databases according to the requirement analysis.

Software	R1				R2					R3							R4		R5		
	Integrated pathway	Variety (coverage) of pathway	Availability of organism-specific pathway maps	Original pathway map/generate by genome	Keyword search	Search by sequence homology	Search by molecular weight	Route search	Links to external DB	Microarray data	Proteome data	Metabolome data	Color code	Multiple conditions and time-series data	Adding annotation by text	Adding drawings and diagrams	Export to BioPAX or SBML	Save as image file	Installation-free	Free for academics	No registration
This work	•		•	•	•	•	•	•	•	•	•	•	•	•	•	•		•	•	•	•
ArrayXPath II [10]			○						•	•			•	•		•		•	•	•	•
BioCarta *1		•	○		•				•							○		•	•	•	•
BioCyc [21]	•	•	•		•	•	•		•	○	•	•	•	○			•	•	•	•	•
BioCyc: Pathway Tools Software [20]	•	•	•	•	•	•	•	•	•	○	•	•	•	○	•	•	•	•		•	
ExPASy Biochemical Pathway [34]	•	○			•				•										•	•	•
GenMAPP [11]		•	○	○	•				•	•	•		•	○			•	•		•	
Genome Projector [25]	•		○	○	•	•			•	○	•		•						•	•	•
Ingenuity Pathway Analysis *2		•	○	○	•				•	•	•	•	•	•	•	•	•	•	•		
iPath [18]	•		•						○	○	•	•	•					•	•	•	•
KEGG [15]	•	•	•	•	•	•		•	•	○	•	•	•				•	•	•	•	•
KEGG Atlas [17]	•		•		•				•									•	•	•	•
MEGU [12]	○	•	•	○					•	•	•	•	•					•	•	•	•
PathCase [44]		•	•	○	•				•							•	•	•	•	•	•
Pathway Explorer [13]		•	○		•				•	•	•		•					•	•	•	•
Reactome [19]	•	•	○		•			•	•	○	•		•				•	•	•	•	•
Reactome β [19]	○	•			•				•										•	•	•
VANTED [45]		•	•	•	•					•	•	•	•	•	•	•	•	•		•	•
WikiPathways [46]		•	○	○	•											•		•	•	•	○

This table summarizes the functions of existing pathway tools according to the requirements identified in this work: (R1) pathway representation, (R2) data access, (R3) mapping and editing, (R4) data export and exchange, and (R5) availability. Closed circles indicate satisfactory implementations, and open circles represent partial implementations.

*1 http://www.biocarta.com/.

*2 http://www.ingenuity.com/.

#### (R1) Pathway representation

A biochemical pathway is often subdivided into smaller maps harboring specific biological processes, such as glycolysis, the TCA cycle, and the pentose phosphate pathway. Capturing the large systematic picture of cellular dynamics that spans several of these specific pathways, however, is difficult, especially in light of the availability of large-scale, comprehensive experimental data from high-throughput “-omics” measurements that encompass various pathways. An integrated pathway map that connects all subdivided pathways into a single large pathway is more suitable and intuitive as a context for information mapping rather than rotating through hundreds of specific maps.

While several pathway components are conserved among different organisms, each species also has its own specific genes, metabolites, and enzymes, and therefore has some unique set of pathways. While a reference pathway map with all known pathway components regardless of the organism, such as the one that is available in the KEGG database, may be a useful gateway, a researcher focusing, for example, on *Escherichia coli* is primarily interested in the pathways of that organism, which makes all the other components dispensable. The availability of organism-specific pathway maps is therefore essential for this purpose, as well as for comparative studies among different organisms. Furthermore, it is desirable for a user to be able to reconstruct a pathway map from his/her own genomic sequence to keep up with the rapid availability of new genomes, both for comparative study and for the functional annotation of the genome.

Although the majority of existing pathway maps belong to the metabolic pathways, numerous aspects of cellular processes are formulated as pathways, including signal transduction, genetic information processing and maintenance, gene regulation, and the cell cycle. The availability of a drug resistance pathway map, for example, will facilitate drug discovery.

#### (R2) Data access

A pathway is a gateway to a wide variety of biological information, including genomic sequences, the functional annotations of genes, the biochemistry of enzymatic reactions, and the chemistry of metabolites. A pathway browser should, therefore, provide links to associated data in external public databases such as PathCase [44]. Various search capabilities are also essential for such highly complex and large-scale information in addition to simple query types, such as keywords and identifiers. The primary outcome from genomic, transcriptomic, and proteomic experiments is the sequence information, which demands sequence similarity searches that incorporate nucleotide or amino acid sequences. Likewise, metabolomic experiments produce spectrograms with molecular mass and retention times, from which corresponding compounds need to be identified.

When observing experimental data in the context of pathways, a biologist is often interested in a subset path or route within a complex pathway map. For example, in gene knockout experiments, biologists are often interested in the change in flux or gene expression within a given route as well as in the activation of alternative paths. Hence, computation and searches for possible routes between two given components is desirable for the observation of alternative paths and for the prediction of unidentified intermediate molecules that are difficult to measure by standard means.

#### (R3) Mapping and editing

An essential feature of a pathway browser is the ability to map and edit experimental data, which can involve changing the colors, sizes, and shapes of pathway graphics according to the experimental data or placing graphs of data onto the locations of respective entities, to visualize the data in the context of the biochemical pathway. Since high-throughput measurement technologies are available, large-scale experimental data from transcriptomics, proteomics, and metabolomics should be supported. For this reason, molecular components, including genes, proteins, enzymes, and metabolites, should be represented and be available for mapping within the pathway map. While genes, proteins, and enzymes can usually be represented as single nodes or sometimes simply as edges in most existing pathway maps, heteromeric enzymes, which are formed from several proteins and, therefore, from several genes, require dedicated nodes to correctly map data from microarray experiments. Furthermore, experimental measurement is often performed on multiple conditions or in a time series, which requires the simultaneous mapping of multiple data to observe the changes and differences. These can be visualized, for example, by using animations that show pathway changes according to time as in the Omics Viewer [20] or by displaying graphs on each object as in VANTED [45].

Naive mapping that is based upon a predefined pathway map does not allow novel pathways or entities to be mapped, which limits the applicability of a pathway browser to evolving knowledge in molecular biology. For example, noncoding RNAs, as typified by micro RNAs (miRNA) or small nucleolar RNAs (snoRNA), or phosphorylated isoforms of proteins are emerging areas of research that are actively being explored and could benefit from interpretation within a biochemical context. Therefore, the pathway map should be able to be freely edited and annotated by adding nodes, edges or data, as in WikiPathways [46].

#### (R4) Data export and exchange

For interoperability and data management, pathway data and mapping results should be downloadable in a standard XML image format, such as SBML (Systems Biology Markup Language) [47] or BioPAX (http://www.biopax.org).

#### (R5) Availability

In order to be platform-independent and interoperable without maintenance and installation efforts, a pathway browser should be available as a web-based application that requires no registration fees and that is freely available for academic users.

### System Overview

**Figure 1:**
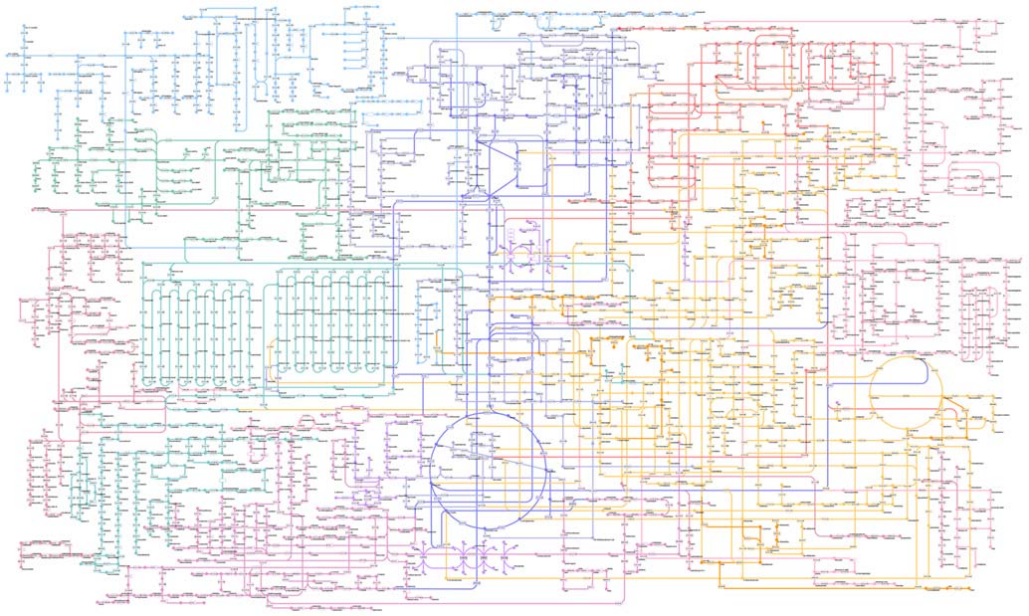
Reference pathway map. Pathway Projector provides an integrated pathway map that is based upon the KEGG Atlas, with the addition of nodes for genes and enzymes. Circles represent metabolites, and rectangles represent enzymes that are further subdivided into several compartments indicating the composite genes for heteromeric enzymes. Nodes are labeled with names or EC numbers at high zoom levels.

**Figure 2:**
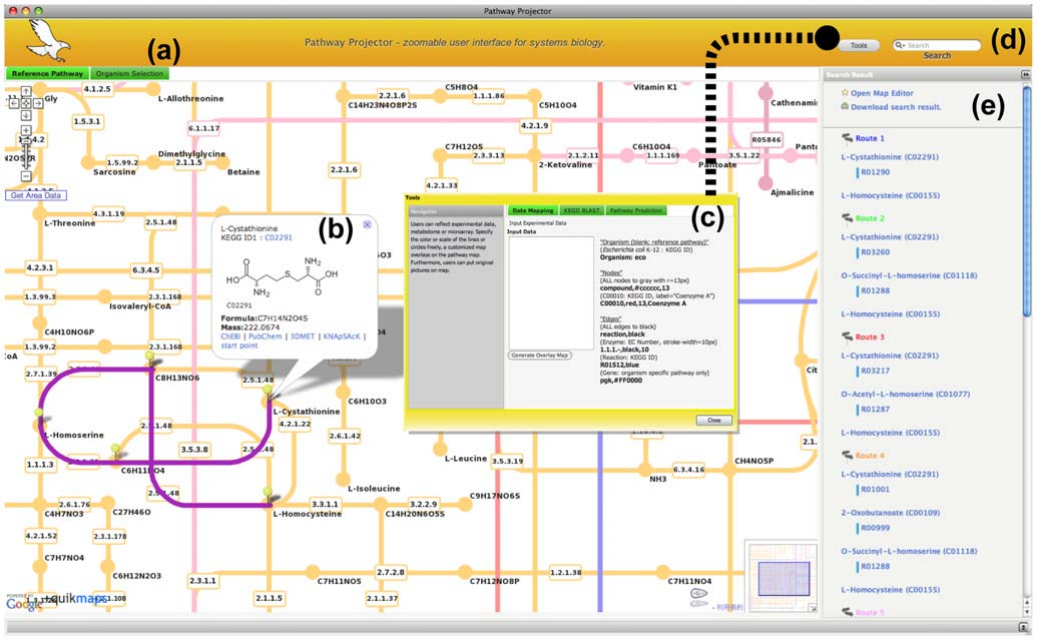
User interface. (a) Organism selection tab lists all available organism-specific pathways, which are opened as new tabs upon selection. (b) The information window is opened by clicking on the entities represented in the map or on the markers that are shown as search results. This window shows detailed information about the selected entity, including names, images of structures, and molecular weight, and provides links to external databases. Furthermore, the selection of two metabolites as starting and ending compounds through this window results in the computation of possible paths between the two selected compounds. The result of path search is displayed in the right-most result panel and as highlighted lines on the map. (c) Data mapping, sequence similarity searches, and pathway reconstructions based on sequence data, are available in a pop-up window that can be invoked from the “Tools” button. (d) The search box located in the top-right corner automatically interprets the given query type and searches accordingly based on keywords, molecular mass, or identifiers. (e) This panel displays the search results as a list. Users can locate the entities by opening an information window, which automatically moves the map to show the selected object in the center. Links to downloadable pathway images and editing and annotation palettes are located above the search results.

The Pathway Projector was implemented according to the aforementioned requirements, including the availability of a large-scale comprehensive pathway map, pathways from a wide variety of organisms, and searching and mapping capabilities. The software is freely available for academic users without any registration at http://www.g-language.org/PathwayProjector/. Since it has been implemented as a web application, this software is cross-platform and requires no installation or maintenance. Moreover, use of the AJAX web development paradigm provides an intuitive user experience similar to that of desktop applications. The main pathway map of Pathway Projector was reconstructed from the popular KEGG Atlas layout by adding nodes for enzymes and genes. As an example, the map for *E. coli* K12 contains 1365 genes, 1813 enzymes, and 1572 metabolites (Figure 1). Circular and rectangular nodes represent metabolites and genes/enzymes, respectively, and the names of genes, enzymes, and metabolites are displayed within the map at high zoom levels by means of semantic zooming. Reaction edges are color coded according to the following pathway categories: aqua represents glycan biosynthesis and metabolism, blue represents carbohydrate metabolism, green represents lipid metabolism, red represents nucleotide metabolism, purple represents energy metabolism, yellow represents amino acid metabolism, pink represents metabolism of cofactors and vitamins, dark red represents biosynthesis of secondary metabolites, orange represents metabolism of other amino acids, and magenta represents biodegradation and metabolism of xenobiotics. Pathway Projector utilizes the Google Maps API for the implementation of ZUI and enables smooth navigation through panning and zooming without page transitions using a mouse scroll wheel or double clicks. Every component in the pathway map shows more detailed information by clicking on the nodes, which opens up an information window containing annotations, such as chemical and structural formulas and links to external public databases (Figure 2b). Detailed information about the nodes can be alternatively accessed through “Mouse Over Mode” that can be toggled from the right-most search result panel, with which users can simply move the mouse over to the nodes to show information in the sub-window located in the bottom panel. Users can therefore use Pathway Projector as a generic browser and as a gateway for various pathway-related resources.

The default pathway map of Pathway Projector is the reference pathway map, but organism-specific pathway maps can be selected from a list of 870 organisms available from the Organism Selection tab located in the upper left section of the user interface (Figure 2a). This list of organisms can be searched incrementally or can be sorted by species names, domains, kingdoms, and sub-phylums by clicking on the column headers. An organism-specific map is opened as a closable tab next to the reference pathway map tab upon double clicking on the desired row in the list. These maps show gene names within enzyme nodes, and the information window also contains organism specific database links, such as EcoCyc for *E. coli* [48] and SGD for yeast [49]. Since tab-browsing has been adopted as an effective approach in navigating the World Wide Web, as seen in numerous web browsers with this function, tab-browsing was utilized for the navigation of different organism-specific maps, which allows the user to quickly switch between species for comparative study.

### Search and Retrieval

Users can search through the pathway components from the search box located in the upper right corner (Figure 2d) using keywords and identifiers for genes, enzymes, pathways, and metabolites, or with the molecular mass of metabolic compounds (default range within **±**10 mass number). Search results are directly shown within the pathway map, marked with red pins on the nodes as well as gray lines highlighting the corresponding edges for reactions. A list of search results is also available in the right-most panel (Figure 2e), where the search range for molecular mass can be adjusted. Clicking on a marker or an entry in the result list invokes an information window.

In order to identify the paths or the existence of alternative pathways, possible routes between two metabolites can be searched in Pathway Projector. Starting and ending metabolites for the route search can be selected from within the information window. The search results are displayed as lists in the search result panel, similar to keyword searches, and routes are highlighted after clicking on the route number. This feature is especially useful when observing the change in the flux distribution upon gene knockout or over-expression experiments to identify the existence of alternative pathways or for the prediction of the concentration of immeasurable metabolites from the changes in neighboring compounds.

A sequence similarity search using nucleotide or amino acid sequences has been implemented based upon KEGG BLAST and is displayed in a pop-up window that is opened by clicking on the “Tools” button located next to the search box (Figure 2c). The system automatically interprets the type of sequence (nucleotide or protein) and subsequently chooses the appropriate program (blastn, blastp, blastx, tblastx); therefore, the user only needs to paste in a sequence of interest to the text area and choose the type of database to run the BLAST search. The search results are marked with highlighted edges on the reference pathway and are also listed in the search result panel with KEGG Orthology identifiers, species names, e-values, and links to organism-specific pathway maps with the BLAST result. The “Pathway Prediction” tab, which is another tool that can be found in the “Tools” window, reconstructs the pathway from given multi-FASTA amino acid files and draws the resulting pathway map accompanied by corresponding e-values. Users can use this feature to analyze novel organisms that are not included in Pathway Projector or to analyze pathways in any given gene set, such as those included in an environmental metagenome database.

### Pathway Mapping of Experimental Data

**Figure 3:**
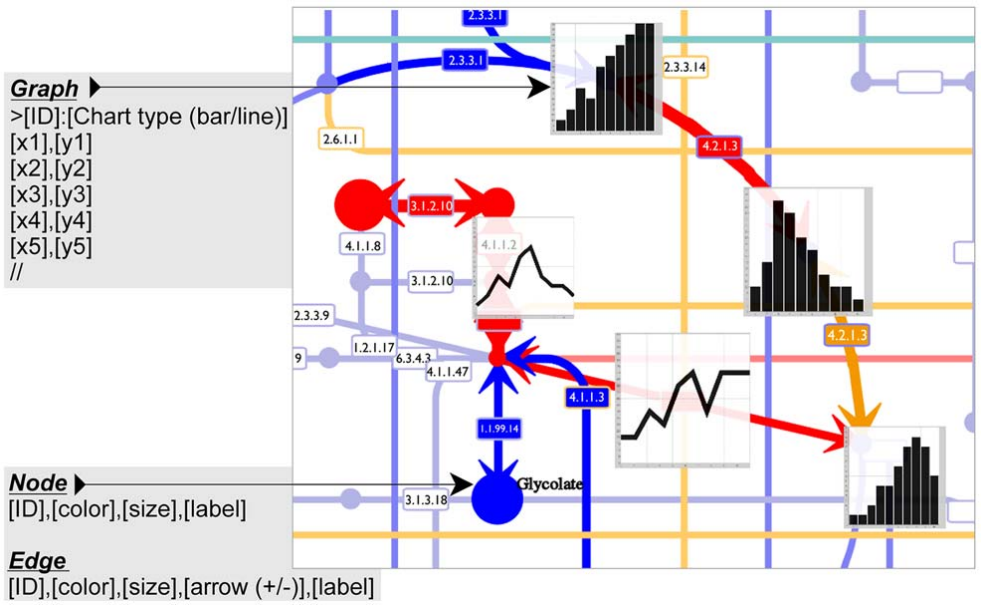
Data mapping. An example result and required data formats for mapping are shown. For graph mapping, the target compound ID and chart type should be defined with a colon “>ID:line”. The graph data is written on the next line and “//” is placed on the final line. For node or edge mapping, user-generated data can be input line-by line following such order by comma, ID, color, size, arrow, and label. Furthermore, details of the graph picture are shown when the graph is clicked.

The mapping feature of Pathway Projector is available from the “Tools” window, which allows full customization of pathway diagrams by changing the color, size or width, labels, and node image (specified by preset icons or URLs of images). In addition, directionality can be indicated by arrow heads, and graphs of multiple conditions or time series can be displayed (Figure 3). Users can map data from transcriptomic, proteomic, and metabolomic experiments, and multiple “-omics” data can be simultaneously represented on a single map. Because the node representing an enzyme is subdivided into multiple compartments when the enzyme is heteromeric and, therefore, comprised of several genes, transcriptome data can be correctly mapped onto individual genes. Entities are specified using the KEGG identifier (*e.g.*, C00010), EC number (*e.g.*, 1.1.1.1), and gene names, while the basal pathway map is specified by the KEGG Organism identifier (*e.g.*, “eco” for *E. coli* K12). The graph of time-series or multi-condition data can also be visualized on top of the corresponding node by specifying the values as comma-separated vectors, and the graphs can be viewed at higher resolution by invoking the information window. Users can also alternatively upload a tab-delimited data file, where the first rows are component identifiers, and the columns are experimental data. By placing these graphs on the metabolic pathway, researchers can easily interpret complex multiple experiments in the context of biochemical pathways and subsequently identify the systematic response to perturbations. Map generation for Google Maps generally requires several minutes of computation time since thousands of tiled images must be prepared on the server; however, through multi-thread and multi-core optimization, Pathway Projector is able to generate the mapped image as Google Maps ZUI in less than 20 seconds on a dual quad-core CPU node. The mapped image is available as a downloadable image or as a transparent overlay, which can be toggled to display by pressing on the “Customized” button located in the upper right corner of the map. Because the mapping feature can specify the basal organism map and because it is implemented as an overlay of existing pathway maps, users can take advantage of this mapping feature for cross-species comparative study. For example, to compare the pathway maps of human and mouse, creating an overlay of mouse maps, which can be simply accomplished by creating a mapping layer with “Organism:mmu”, on top of the human map allows rapid switching between the two pathways. Simple Object Access Protocol (SOAP) web service is also available for data mapping for interoperability with other tools. Users can access this service through programming languages such as Perl, Python and Ruby, or through intuitive SOAP clients with graphical user interfaces such as Taverna workbench [50]. Web Service Description Language (WSDL) file is available at http://www.g-language.org/PathwayProjector/PathwayProjector.wsdl and detailed documentation as well as sample scripts for several programming languages are available in the online documentation (http://www.g-language.org/PathwayProjector/annotation.html#soap).

**Figure 4:**
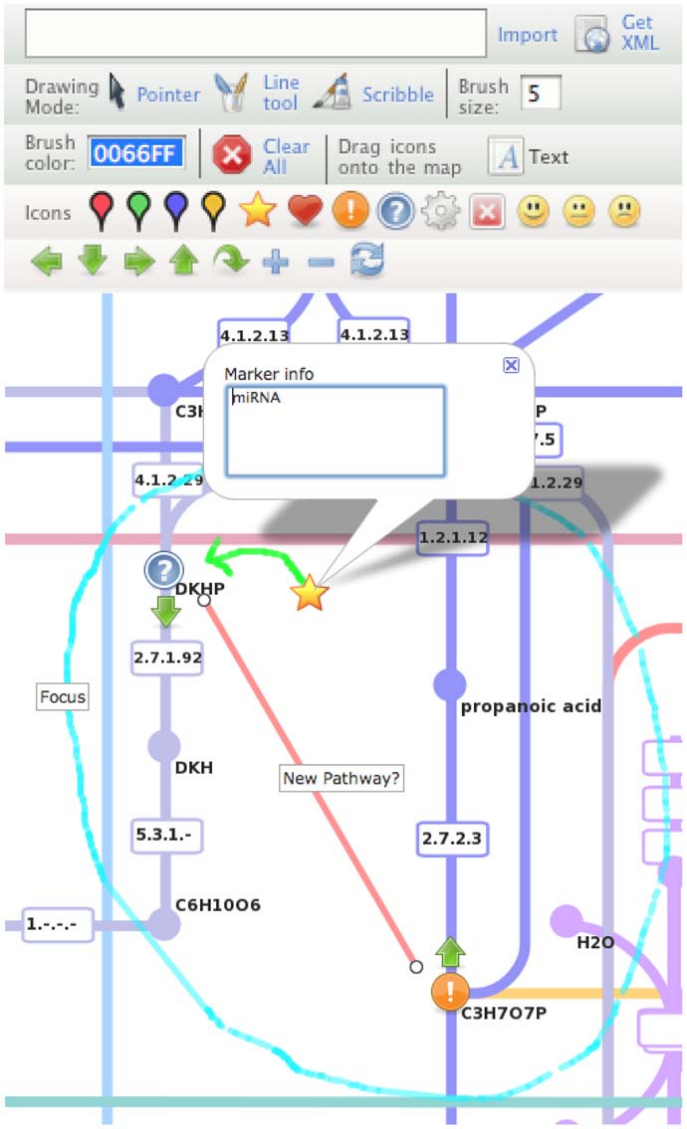
Manual editing and annotation of pathway maps. The pathway editing and annotation palette can be invoked from the link located at the top of the search results panel. Twenty-one pre-set icons are available to be dragged and dropped anywhere in the map, functioning as original markers. Users can freely move these markers around the map and can also add annotations and comments by clicking on the markers. Several other drawing features are available, including the line tool for drawing lines, scribble tool for free drawing, and text tool for placing labels. Brush color and size can be customized. Users can export the edited map as an XML file from the “Get XML” button, and this file can be shared and imported by other users to recapture the edited map.

Pathway Projector is also equipped with editing capabilities, such as drawing lines, placing icons and text labels, and adding annotations, to add novel findings that are not included in the reference pathway (Figure 4). The editing tool is opened by clicking on the “Open Map Editor” link in the right-most panel. The line tool and scribble tool are available for drawing lines and curves, respectively, and the brush size and color for these tools can be configured. Text labels and icons can be dragged and dropped onto the map, and these objects can be clicked to show an editable information window in which the users can add custom annotations. The edited and annotated pathway map data are downloadable as XML files that can be saved and exchanged. This XML file is based on the Quikmaps format, and contains the editing log including the texts, coordinates, color and size of lines or icons. Hence, users can recapture the manually edited map by pasting this XML log and then clicking on the “import” button in a new session. This XML file can be shared among researchers to share the manual annotations.

### Limitations

Pathway Projector is currently limited to the metabolic pathway, due to the availability of the global map in KEGG Atlas. Therefore, pathways such as signaling networks, gene regulation, and cell cycle are not supported by our software. Since our software tool is semi-automated to import information from KEGG, large-scale maps for these particular pathways, if they become available, will be relatively easy to integrate into Pathway Projector. Second, the KEGG pathway maps are generic implementations, for which the reference pathway serves as the net sum of all known reactions from a multitude of organisms. There are cases for which a specific pathway map dedicated to just a single species of interest is instead more desirable, such as the PMN (http://www.plantcyc.org/) for plants, EcoCyc for *E. coli*, and MGD [51] for mouse. Although links are provided to these databases for organism-specific information, we may need to consider using organism-specific maps for well-curated pathways in addition to those of KEGG, when integrated pathways become available in these websites. Nevertheless, the basic user interface frameworks based on the AJAX technology for searching and mapping, as well as the ZUI based on Google Maps API, are applicable for other pathway maps as long as the coordinate information is provided along with the map image. Finally, Pathway Projector does not provide pathway data in an exchangeable format such as SBML and BioPAX, as noted in the requirement analysis section. However, since our system is based on the KEGG maps, KGML (KEGG Markup Language) can be readily converted into the BioPAX format, and these BioPAX file can be downloaded from ftp://ftp.genome.jp:/pub/db/community/biopax. The map editing features provided in this software is intended for quick note taking for information exchange along researchers. While this feature allows free editing and annotations on the pathway maps, these editings are overlays of additional information, and are not true modifications to the existing pathway map. For the generation of static pathway map images, users should use the mapping tool to generate a custom pathway map, and download this image to make permanent modifications.

### Conclusion

In light of the advent of high-throughput measurement technologies among several layers of “-omics,” understanding the systematic workings of the intracellular activities is critical, and biochemical pathways provide an indispensable context for this purpose. Since pathways are essentially connected *in vivo*, the use of a global map is desirable, especially for the mapping of comprehensive experimental data. Pathway Projector is an intuitive browser that has been designed for this purpose by providing a large-scale metabolic pathway map based on the popular KEGG Atlas layout, with the addition of gene and enzyme nodes. Pathway Projector has been implemented with user-friendly interfaces while also providing its software as web-based application for cross-platform availability. Integrated search capabilities, as well as mapping and editing capabilities, facilitate exploratory and heuristic data analysis. Therefore, Pathway Projector can serve as a useful gateway for pathway information analysis.

## Supporting Information

Table S1 List of supported organism-specific external databases.
(0.08 MB DOC)
