# Supplementary material for: Pathway Projector: Web-Based Zoomable Pathway Browser Using KEGG Atlas and Google Maps API
Source: PLoS One. 2009 Nov 11;4(11):e7710. doi: 10.1371/journal.pone.0007710 (PMC2770834; doi:10.1371/journal.pone.0007710)
Supplement: Table S1 — List of supported organism-specific external databases. (0.08 MB DOC) [file pone.0007710.s001.doc]

**Table S1**

| **Databases** | **References (or URL)** | **Databases** | **References (or URL)** |
| --- | --- | --- | --- |
| CryptoDB | [1] | MBR | [26] |
| BROAD | [2] | MGI | [27] [28] |
| Bordeaux | [3] | MIPS | [29] |
| British Columbia | [4] | MypuList | [30] |
| FusionDB | [5] | NCBI | [31] |
| CYORF | [6] | NITE | [32] |
| Cambridge | [7] | OMIM | [31] |
| DictyBase | [8] | PDB | [33] |
| EcoCyc | [9] | PROSITE | [34] |
| EcoGene | [10] | Pathema | [35] |
| Ensembl | [11] | PathoGenesis | [36] |
| FlyBase | [12] | Pfam | [37] |
| GeneDB | [13] | PlasmoDB | [38] |
| Genoscope | [14] | RATMAP | [39] |
| HGNC | [15] | RGD | [40] |
| HPRD | [16] | RegulonDB | [41] |
| IMGT | [17] | SGD | [42] |
| CyanoBase | [18] | SagaList | [43] |
| Kitasato | [19] | ShiBASE_China | [44] |
| LANL | [20] | TAIR | [45] |
| LNCC | [21] | TGD | [46] |
| UniProt | [22] | WormBase | [47] |
| Unicamp | [23] | Xenbase | [48] |
| VCU | [24] | ZFIN | [49] |
| VectorBase | [25] | InterPro | [50] |

1. Heiges M, Wang H, Robinson E, Aurrecoechea C, Gao X, et al. (2006) CryptoDB: a Cryptosporidium bioinformatics resource update. Nucleic Acids Res 34: D419-422.
2. http://www.broadinstitute.org/cgi-bin/annotation/microbes/mycoplasma_mobile/findfeatures.cgi
3. Sherman DJ, Martin T, Nikolski M, Cayla C, Souciet JL, et al. (2009) Génolevures: protein families and synteny among complete hemiascomycetous yeast proteomes and genomes. Nucleic Acids Res 37: D550-554.
4. Warren R, Hsiao WW, Kudo H, Myhre M, Dosanjh M (2004) Functional characterization of a catabolic plasmid from polychlorinated- biphenyl-degrading Rhodococcus sp. strain RHA1. J Bacteriol 186: 7783-7795.
5. Suhre K, Claverie JM (2004) FusionDB: a database for in-depth analysis of prokaryotic gene fusion events. Nucleic Acids Res 32: D273-276.
6. Sugita C, Ogata K, Shikata M, Jikuya H, Takano J, et al. (2007) Complete nucleotide sequence of the freshwater unicellular cyanobacterium Synechococcus elongatus PCC 6301 chromosome: gene content and organization. Photosynth Res 93: 55-67.
7. Oliynyk M, Samborskyy M, Lester JB, Mironenko T, Scott N, et al. (2007) Complete genome sequence of the erythromycin-producing bacterium Saccharopolyspora erythraea NRRL23338. Nat Biotechnol 25: 447-453.
8. Chisholm RL, Gaudet P, Just EM, Pilcher KE, Fey P, et al. (2006) dictyBase, the model organism database for Dictyostelium discoideum. Nucleic Acids Res 34: D423-427.
9. Keseler IM, Bonavides-Martínez C, Collado-Vides J, Gama-Castro S, Gunsalus RP, et al. (2009) EcoCyc: a comprehensive view of Escherichia coli biology. Nucleic Acids Res 37: D464-470.
10. Rudd KE (2000) EcoGene: a genome sequence database for Escherichia coli K-12. Nucleic Acids Res 28: 60-64.
11. Hubbard TJ, Aken BL, Ayling S, Ballester B, Beal K, et al. (2009) Ensembl 2009. Nucleic Acids Res 37: D690-697.
12. Tweedie S, Ashburner M, Falls K, Leyland P, McQuilton P, et al. (2009) FlyBase: enhancing Drosophila Gene Ontology annotations. Nucleic Acids Res 37: D555-559.
13. Hertz-Fowler C, Peacock CS, Wood V, Aslett M, Kerhornou A, et al. (2004) GeneDB: a resource for prokaryotic and eukaryotic organisms. Nucleic Acids Res 32: D339-343.
14. Jaillon O, Aury JM, Noel B, Policriti A, Clepet C, et al. (2007) The grapevine genome sequence suggests ancestral hexaploidization in major angiosperm phyla. Nature 449: 463-467.
15. Bruford EA, Lush MJ, Wright MW, Sneddon TP, Povey S, et al. (2008) The HGNC Database in 2008: a resource for the human genome. Nucleic Acids Res 36: D445-8.
16. Keshava Prasad TS, Goel R, Kandasamy K, Keerthikumar S, Kumar S, et al. (2009) Human Protein Reference Database--2009 update. Nucleic Acids Res 37: D767-772.
17. Lefranc MP, Giudicelli V, Ginestoux C, Jabado-Michaloud J, Folch G, et al. (2009) IMGT, the international ImMunoGeneTics information system. Nucleic Acids Res 37: D1006-1012.
18. Nakamura Y, Kaneko T, Tabata S (2000) CyanoBase, the genome database for Synechocystis sp. strain PCC6803: status for the year 2000. Nucleic Acids Res 28: 72.
19. Ikeda H, Ishikawa J, Hanamoto A, Shinose M, et al. (2003) Complete genome sequence and comparative analysis of the industrial microorganism Streptomyces avermitilis. Nat Biotechnol 21: 526-531.
20. <http://www.oralgen.lanl.gov/oralgen>
21. Vasconcelos AT, Ferreira HB, Bizarro CV, Bonatto SL, Carvalho MO, et al. (2005) Swine and poultry pathogens: the complete genome sequences of two strains of Mycoplasma hyopneumoniae and a strain of Mycoplasma synoviae. J Bacteriol 187: 5568-5577.
22. The UniProt Consortium (2009) The universal protein resource (UniProt) 2009. Nucleic Acids Res 37: D169-174.
23. de Silva AC, Ferro JA, Reinach FC, Farah CS, Furlan LR, et al. (2002) Comparison of the genome of two Xanthomonas pathogens with differing host specificities. Nature 417: 459-463.
24. Xu P, Widmer G, Wang Y, Ozaki LS, Alves JM, et al. (2004) The genome of Cryptosporidium hominis. Nature 431: 1107-1112.
25. Megy K, Hammond M, Lawson D, Bruggner RV, Birney E, et al. (2009) Genomic resources for invertebrate vectors of human pathogens, and the role of VectorBase. Infect Genet Evol 9: 308-313.
26. [http://mycoplasma.genome.uab.edu](http://mycoplasma.genome.uab.edu/)/
27. International Mouse Knockout Consortium, Collins FS, Rossant J, Wurst W. (2007) A mouse for all reasons. Cell 128: 9-13.
28. Collins FS, Finnell RH, Rossant J, Wurst W. (2007) A new partner for the international knockout mouse consortium. Cell 129: 235.
29. Mewes HW, Dietmann S, Frishman D, Gregory R, Mannhaupt G, et al. (2008) MIPS: analysis and annotation of genome information in 2007. Nucleic Acids Res 36: D196-201.
30. Chambaud I, Heilig R, Ferris S, Barbe V, Samson D, et al. (2001) The complete genome sequence of the murine respiratory pathogen Mycoplasma pulmonis. Nucleic Acids Res 29: 2145-2153.
31. Sayers EW, Barrett T, Benson DA, Bryant SH, Canese K, et al. (2009) Database resources of the National Center for Biotechnology Information. Nucleic Acids Res 37: D5-15.
32. <http://www.bio.nite.go.jp/dogan/>
33. Henrick K, Feng Z, Bluhm WF, Dimitropoulos D, Doreleijers JF, et al. (2008) Remediation of protein data bank archive. Nucleic Acids Res 36: D426-433.
34. Hulo N, Bairoch A, Bulliard V, Cerutti L, Cuche BA, et al. (2008) The 20 years of PROSITE. Nucleic Acids Res 36: D245-249.
35. Greene JM, Collins F, Lefkowitz EJ, Roos D, Scheuermann RH, et al. (2007) National Institute of Allergy and Infectious Diseases bioinformatics resource centers: new assets for pathogen informatics. Infect Immun 75: 3212-3219.
36. Winsor GL, Van Rossum T, Lo R, Khaira B, Whiteside MD, et al. (2009) Pseudomonas Genome Database: facilitating user-friendly, comprehensive comparisons of microbial genomes. Nucleic Acids Res 37: D483-488.
37. Finn RD, Tate J, Mistry J, Coggill PC, Sammut SJ, et al. (2008) The Pfam protein families database. Nucleic Acids Res 36: D281-288.
38. Aurrecoechea C, Brestelli J, Brunk BP, Dommer J, Fischer S, et al. (2009) PlasmoDB: a functional genomic database for malaria parasites. Nucleic Acids Res 37: D539-543.
39. Petersen G, Johnson P, Andersson L, Klinga-Levan K, Gómez-Fabre PM, et al. (2005) RatMap--rat genome tools and data. Nucleic Acids Res 33: D492-494.
40. Twigger SN, Shimoyama M, Bromberg S, Kwitek AE, Jacob HJ, et al. (2007) The Rat Genome Database, updata 2007--easing the path from disease to data and back again. Nucleic Acids Res 35: D658-662.
41. Gamma-Castro S, Jiménez-Jacinto V, Peralta-Gil M, Santos-Zavaleta A, Peñaloza-Spinola MI, et al. (2008) RegulonDB (version 6.0): gene regulation model of Escherichia coli K-12 beyond transcription, active (experimental) annotated promoters and Textpresso navigation. Nucleic Acids Res 36: D120-4.
42. Hong EL, Balakrishnan R, Dong Q, Christie KR, Park J, et al. (2008) Gene Ontology annotations at SGD: new data sources and annotation methods. Nucleic Acids Res 36: D577-581.
43. Lechat P, Hummel L, Rousseau S, Moszer I (2008) GenoList: an integrated environment for comparative analysis of microbial genomes. Nucleic Acids Res 36: D469-474.
44. Yang J, Chen L, Yu J, Sun L, Jin Q (2006) ShiBASE: an integrated database for comparative genomics of Shigella. Nucleic Acids Res 34: D398-401.
45. Swarbreck D, Wilks C, Lamesch P, Berardini TZ, Garcia-Hernandez M, et al. (2008) The Arabidopsis Information Resource (TAIR): gene structure and function annotation. Nucleic Acids Res 36: D1009-1014.
46. Stover NA, Krieger CJ, Binkley G, Dong Q, Fisk DG, et al. (2006) Tetrahymena Genome Database (TGD): a new genomic resource for Tetrahymena thermophila research. Nucleic Acids Res 34: D500-503.
47. Rogers A, Antoshechkin I, Bieri T, Blasiar D, Bastiani C, et al. (2008) WormBase 2007. Nucleic Acids Res 36: D612-617.
48. Bowes JB, Snyder KA, Segerdell E, Gibb R, Jarabek C, et al. (2008) Xenbase: a Xenopus biology and genomics resource. Nucleic Acids Res 36: D761-767.
49. Sprague J, Doerry E, Douglas S, Westerfield M (2001) The Zebrafish Information Network (ZFIN): a resource for genetic, genomic and developmental research. Nucleic Acids Res 29: 87-90.
50. Hunter S, Apweiler R, Attwood TK, Bairoch A, Bateman A, et al. (2009) InterPro: the integrative protein signature database. Nucleic Acids Res 37: D211-215.
